# Online Food Shopping: A Conceptual Analysis for Research Propositions

**DOI:** 10.3389/fpsyg.2020.583768

**Published:** 2020-09-16

**Authors:** Chi-Fang Liu, Chien-Ho Lin

**Affiliations:** Department of Business Administration, Cheng Shiu University, Kaohsiung, Taiwan

**Keywords:** online food shopping, conceptual analysis, future research, propositions, theory and practice

## Abstract

Shopping foods online is different from shopping other things online. To stimulate more thinking and enrich potential future research imagination, this paper reviews for online food shopping features, offers a commentary, and proposes future research directions. The propositions include the following: (1) The design and implementation of online food shopping (eco)systems should engage the consumers and other stakeholders to co-create collective and social values; (2) A better fit between technologies’ and food businesses’ natures could generate better applications for online food shopping; (3) A business model with sound finance systems becomes the core of a healthy online food ecosystem; (4) The interaction and transformation between online (virtual) and offline (virtual) food businesses determines the dynamic development of future food shopping.

## Introduction

Most studies on online shopping focus on the implications and benefits of e-commerce. This focus is expected to increase as more people are pushed toward shopping online in a bid to avoid crowded shopping malls for fear of contracting the dreaded COVID-19 virus. A gap in the literature, however, is that while the topic is rife with studies detailing how online shopping works, there is limited research on shopping foods online, which is inherently with very different characteristics from buying other kinds of commodities via the World Wide Web. Nonetheless, food is one of the most common products for the mankind, and so are with great impact for human’s online shopping life. A critical analysis for in-depth understanding of the special attributes that online food shopping has can facilitate the construction of a precise (for stakeholders’ needs) and high-quality (for stakeholders’ safety and satisfactions) online food shopping ecosystem. This paper presents a conceptual analysis aimed at explicating the significant themes within the current literature. The review will conduct critical propositions reflected from these studies to propose future research directions. The academic review is significant to both researchers and online food stores as people across the world start embracing online shopping more than ever before.

## Background Descriptions

Before beginning the conceptual analysis with literature review, a broader background discussion is needed. Practically, the broader background constitutes: e-commerce platforms, consumer preferences and attitudes, marketing approaches, and packaging and delivery considerations.

### E-Commerce Platforms

Silva et al. (2017) define e-commerce platforms as the set of technologies designed to help online businesses to manage their marketing, sales, and operations. Wei’s et al. (2018) study sought to examine the purchase intention of fruits among online shoppers. The authors argue that the past few years have seen the emergence of online purchase platforms for fruits, a trend that has significantly advanced e-commerce development and improved the quality of human life. Although their study sought to investigate consumers’ purchase intention, the results reveal that compared to other products, the e-commerce platforms for fruits did not play a major role in influencing a buyers’ purchase decision. On the contrary, the success of fashion products and electronics is dependent on how online customers perceive their e-commerce platforms (Huete-Alcocer, 2017). For example, customers are less likely to purchase luxury fashion products from a poorly designed website (Kang et al., 2020) and (Buckley, 2016). Thus, while there are limited studies on the differences between buying food and other products online, at least the current studies evidence that e-commerce platforms do not play a significant role in influencing buyers’ purchase decisions.

### Consumer Preferences and Attitudes

Kim Dang et al. (2018) study on consumer preference and attitudes regarding online food products examines how the Internet has changed people’s food-buying behaviors. The study is significant because it establishes the underlying consumers’ concerns with regards to food safety information, especially for online food products. Compared to other products, consumer preferences and attitudes toward buying food online differs in that the perceived risks and information quality do not play major roles in influencing their buying behavior (Li and Bautista, 2019; Sanchez-Sabate and Sabaté, 2019; Zieliñska et al., 2020). Kim Dang et al. (2018) study relies on a cross-sectional study conducted in Hanoi, Vietnam. The findings are reliable as they are based on responses gathered from over 1736 customers through face-to-face interviews. While the preferences and attitudes toward buying food online differ from buying other commodities, Kim Dang et al. (2018) note that the laws governing e-commerce in Vietnam are the same. As such, the findings provide practical advice to online food retailers and the Vietnam government on how to implement appropriate legislation with regards to trading online food products.

Martínez-Ruiz and Gómez-Cantó’s (2016) study emphasizes that using the Internet to seek food service information has now become a common practice among people today. More people than ever before have positive attitudes toward finding information about food online (Martínez-Ruiz and Gómez-Cantó, 2016; Maison et al., 2018). Also, people are more likely to search information about food on the Internet than any other product or service (Hidalgo-Baz et al., 2017; Whiley et al., 2017; Wong et al., 2018). However, Kim Dang et al. (2018) study found that a significant number of consumers were unconcerned about the accuracy of the evidence regarding food safety they found online in selecting food products on the Internet. The conclusions drawn from the current article review produces practical pieces of advice to consumers buying food online as well as the food retailers selling food over the Internet.

### Marketing Approaches

Rummo et al. (2020) examine the relationship between youth-targeted food marketing expenditures and the demographics of social media followers. The authors sought to establish the extent to which teenagers follow food brands on Twitter and Instagram by examining the relationships between brands’ youth-targeted marketing practices and the overall percentages of adolescent followers. The study provides evidence showing that unhealthy food brands, especially fast food and sugary drink have more adolescent followers on social media (Rummo et al., 2020). These study results are consistent with Salinas et al. (2014) findings which show that unhealthy food products enjoy a higher market base than the healthy ones. The high percentage of teenage followers is concerning among health experts mainly because most of the advertisements from these companies are biased and do not highlight the unhealthy consequences associated with eating these foods. Compared to other products, food companies are often not required by regulations to highlight their negative consequences (Salinas et al., 2014). For example, cigarette and alcohol companies are mandated to disclose their effects of use on all marketing materials (Gravely et al., 2014). Consequently, with the ubiquitous use of social media by teenagers, young people are more exposed to food and beverage advertising which occurs across multiple digital channels.

The failure to address digital advertising when formulating policies makes it harder to governed youth-targeted food marketing. Food products are often marketed using the general techniques and approaches applied in other products and services. Juaneda-Ayensa et al. (2016) note that food marketing topics such as market segmentation, strategic positioning, test marketing, branding, consumer research, targeting, and market entry strategy are highly relevant. Moreover, food marketing is affected by the major challenges that affect conventional markets such as dealing with perishable products whose availability and quality varies as a function of the current harvest conditions (Hongyan and Zhankui, 2017). However, Topolinski et al. (2015) note that the value chain in food marketing is particularly important because it highlights the extent to which sequential parties within the marketing channel add value to the final product. According to Linder et al. (2018) processing new distribution options often provides additional opportunities available to food marketers to provide the final consumer with convenience. However, when overhead costs such as marketing and processing are added they result in significantly higher costs (Lou and Kim, 2019).

Demographics play an essential role in food marketing almost more than any other product. According to Qobadi and Payton (2017), food companies must utilize statistical demographics to understand the inherent characteristics of a population. For food marketing purposes, such knowledge can help firms develop a better understanding of the current market place as well as predict future trends (Isselmann DiSantis et al., 2017). For example, with regards to the current market, food companies interested in entering a new market with sports drinks might first study the overall number of people between the ages of 15 and 35, who would constitute a particularly significant market. In such cases, most food companies often prefer shifting their resources toward products consumed by a growing population. As such, the success of the marketing strategy employed by a food company is contingent on how good it studies the demographical makeup of its target market.

### Packaging and Delivery Consideration

One of the primary consideration food consumers take into account when making a purchase decision online involves packaging and delivery. According to Chen et al. (2019), the modern consumer is more interested in food products that utilize sustainable packaging and delivery systems. Hu et al. (2019) add that most customers today are more focused on recyclable packaging systems. Grace (2015) further notes that sustainability is one of the primary sustainability attributes online shoppers look for. For example, over 33% of online consumers believe that packaging and recyclability are more important to them when ordering food items online (Gutberlet et al., 2013). Additionally, 13% of online shoppers cite a lack of packaging information available online, which suggests that there is an existing opportunity for e-retailers to increase their sustainability information (Quartey et al., 2015).

As the world continue grappling with the COVID-19 pandemic, online purchases for fresh food is gradually becoming the norm across the world. As such, food producers must be able to adapt accordingly to take advantage of the emerging market. However, the majority of consumers are still concerned about freshness and food waste (Yu et al., 2020). Unlike in a brick-and-mortar store where shoppers can visibly check the freshness of their produce, this is more difficult with home delivery (Song et al., 2016). Thus, brands must try and opt for packaging that can keep food safe and fresh during transit and displays its freshness to re-assure customers. Moreover, to meet sustainability goals, fresh food brands need to balance the use of more sustainable, recyclable materials, with packaging that continues to extend shelf life and avoid food waste.

## Conceptual Analysis for Future Research Propositions

The article review shows that sufficient studies have been conducted on online food shopping. As more people start shopping online, the number of articles on online food shopping is expected to increase. However, despite studies on online food shopping and business models remain rife, there are key gaps in research. These gaps are a result of the majorities of the researchers’ focus on highlighting their perspectives and largely ignore those of the consumers and businesses. Moreover, these studies do not consider crisis (e.g., COVID-19 pandemic) when making these future predictions. The forecasts made about future help in developing a better understanding of the various implications of ordering via mobile apps. Also, it provides a background for examining the emerging technologies in online food ordering. As such, the critical propositions reflected in the literature review propose the following four future research directions.

### Value Co-creation With Stakeholders

From a business perspective, getting partners and investors on board is not easy and most restaurants tend to stay away from technology. Thus, the preposition made involves conducting research aimed at developing a better understanding of the customer and business’ perspectives. According to Chen et al. (2018), setting the commission rates with restaurants is a major problem within the online food industry. Moreover, the majority of startups are depended on restaurants to deliver food at the customer’s doorstep (Onyeneho and Hedberg, 2013). Hwang et al. (2020) argue that relying on technology is not the main focus of a restaurant because preparing food is its main core business. As such, even if an investor trusts a food startup, integrating technology within its business process will always be perceived as a high risk. The lack of sufficient evidence on the business’ perspective toward technology and online platforms make it more difficult for rescuers to tailor their studies to generate crucial insights that help in making better business decisions.

One of the problems identified from the consumer’s perspective is that most of the things mentioned in the online food menus are often not available. Instead, they act as click baits designed to entice online users to continue interacting with their platform and marketing content (Lara-Navarra et al., 2020). In rare cases, some clickbait links often forward online users to pages that require them to make payments, register, or even fill in their payment details. Consequently, a significant communication gap exists between consumers and restaurants while shopping on phone and online. While numerous studies examine the purchase intention of food among online shoppers, few highlight the inherent challenges experienced by consumers as they go about their day.

While it is crucial to investigate both perspectives, more studies need to be conducted on the customer ones. This is because most online businesses often find it difficult to deal with customers, but Ho et al. (2014) note that this is usually because they do not see things from the buyers’ point of view. The authors, however, refutes the popular phrase that “customer is always right” and notes that even when they are completely wrong, they can always win. For example, customers can criticize a business online or even refuse to pay their bills. As such, failing to grasp a customer’s perspective can result in a meltdown with them which is always bad business. It is also essential for future businesses to take into consideration the fact that work is much more enjoyable and profitable when people work alongside the customer rather than against them. Thus, conducting more studies aimed at understanding customers can help develop the necessary recommendations to help businesses see things from their point of view.

One of the ways future studies can explore to better understand the customer’s perspective involves exploring the issues related to empathy. Charles et al. (2018) note that empathy does note naturally to most people but it reinforces one’s ability to understand and share the feelings of a customer by placing themselves in their shoes. Future studies should highlight how online businesses can ask questions about how their current and potential customers would feel in different circumstances. Also, future studies must examine how well online businesses can listen to their customers. Afshar Jahanshahi and Brem (2018) notes that the first step in customer relations involves actively listen to them. Finally, future studies must be able to provide recommendations on how online food businesses can grow trust and show respect to their customers. The prepositions made with regards to the business and customers’ perspective provides the background information for future studies. Also, bridging the current research gaps will help business adopt a more effective online model that maximizes customer satisfaction when purchasing foods. Based on the discussions above, this article suggests the following proposition to both identify the gap in the literature and the corresponding future research directions.

***Proposition 1:*** *the design and implementation of online food shopping (eco)systems should engage the consumers and other stakeholders to co-create collective and social values.*

### Technological Nature

Although smartphone apps provide an efficient way to replace the conventional methods of ordering food through a phone call, there lacks sufficient evidence on the implications of placing orders through them. A partial but potentially important reason is the lack of in-depth and broader understanding of the technology *per se*. Mobile ordering apps have caused a significant change in food delivery and pickup business (Onyeneho and Hedberg, 2013). With more and more retailers and restaurants adopting these technologies, the modern consumer is willing to place fewer delivery and pickup orders through their phones. Instead, they are now opting to utilize mobile apps. Studies aimed at exploring the implications of food delivery apps help in establishing whether it is hurting or assisting the business. Thus, as a restaurant owner, one has to be careful with regards to utilizing third-party services to do business. For instance, apps such as Uber Eats have endless possibilities as they make delivery faster, for both the customers and the business. However, future studies must examine the potential disadvantages to using such third-party services. Firstly, the added cost of a food delivery app may be prohibitive to most customers. For example, the cost of using services like Uber Eats changes how businesses price their meals. In the end, customers are likely to end up paying more. Thus, future studies have to consider this fact when developing recommendations on how businesses can use food delivery apps without undermining their financial positions. Also, these studies will help show how customers are likely to react to a price surges.

Subsequent studies on the implications of ordering food through mobile apps should also focus on the issues relating to control and accountability. Cecchi and Cavinato (2019) note that some customers have complained about being unable to control the food ordering process. For example, once the customer’s food is in the possession of the Uber driver, there is little left for them to do, which is perceived as a bad thing. Also, Isoni Auad et al. (2018) note that customers lack control over how their drivers handle their food. One of the consequences of being unable to control the process is that when a customer’s food is mishandled or ends up late, the restaurant is the one that is held accountable. Finally, with regards to the implications, future studies must monitor their third party service to safeguard their brand’s reputation. As such, subsequent studies need to ensure that they highlight the importance of maintaining an effective brand image. Mao et al. (2018) recommend online food businesses to monitor how long it takes their delivery people to transport their customers’ food to establish whether it is being handled with the necessary care it deserves. However, more studies are required to highlight the customer’s grievance which can easily fall on the businesses when the delivery issues are ignored.

Despite the various implications of using mobile apps to order food online, there are numerous benefits associated with online models. As such, as the growth of online applications continues, the subsequent studies need to add to the existing literature on the benefits businesses are likely to accrue from adopting such technologies. According to Li et al. (2020), this trend is a result of the numerous benefits associated with using the apps compared to the conventional methods of shopping over the phone or waiting in line. These benefits are 2-fold, they include benefits to the consumer and the restaurants. Firstly, there are numerous consumer benefits of using mobile ordering apps to purchase food.

Consumers across the world are downloading mobile ordering apps at lightning speed. For example, When Chick-fil-A, one of the largest American fast food restaurant chains, released its first official app, it reached first place in the app store in only 3 days after it was launched. Mayordomo-Martínez et al. (2019) note that these apps are popular for four main reasons. Firstly, customers feel that no one is waiting in line or getting put on hold. Secondly, customers can pick up food on the go. Thirdly, customers get the whole menu right at their fingertips, including items they may not have known existed. Finally, most restaurants award patrons’ loyalty reward points. In most cases, these points are easy to track directly through applications and lead to big savings if the customer order frequently.

The restaurant benefits from the mobile ordering apps too. While these apps may be created for the customer, they achieve some important objectives that can greatly help out the restaurant or retail store as well (Ferguson and Solo-Gabriele, 2016). For example, they can handle more orders as is the case with Chipotle, an American chain of fast-casual restaurants, which claims that it is capable of processing six additional orders every hour when placed through a mobile app (Ferguson and Solo-Gabriele, 2016). Moreover, customers are more likely to spend more through an ordering app than in person because they have more time to decide since the entire menu is in front of them and they typically want to score more reward points. Based on the discussion above, this article made the propositions as follow.

***Proposition 2:*** *A better fit between technologies’ and food businesses’ natures could generate better applications for online food shopping.*

### New Business Models and Finance Systems

Although numerous studies have highlighted the various emerging trends in buying food online, most were conducted before the COVID-19 pandemic. As such, future studies need to capture how the pandemic has affected the online ordering industry. Such studies will provide the insights necessary to help the business withstand emerging competition as well as keep up with the ever-changing customer demands and the latest trends and technological advancements. Wang et al. (2020) note that the various responses to the COVID-19 global pandemic will shape the online food delivery industry in 2020 and beyond. Thus, future studies need to identify and critically examine the top online food shopping trends that customers and businesses must remain aware of.

For the better part of the year 2020, global cities have become deserted and shopping malls closed. The restaurant sector is one of the most affected as people are recommended to maintain social distancing and remain at home. As the Coronavirus continue spreading across the world, the pandemic is projected to have more economic implications than undermine global health. Thus, future studies must offer people a glimpse of how lockdowns will affect the online food industry, which is hailed as the future in the restaurant sector. However, even at the current stage of the Covid-19 pandemic lifecycle, several lessons are already emerging from China with regards to how people can cope with the commercial and social disruptions. For example, the pandemic is a key driver for digital technologies.

There are three areas that future studies need to focus on. They include the emergence of digitally enabled delivery systems and consumer comfort with the online food sector. Firstly, the prevalence of digitally enabled delivery systems is expected to grow in the coming years. As such, studies are needed to develop a better understanding of how these online delivery systems will affect the food industry. For example, since the COVID-19 pandemic began, more people than ever before purchase their groceries and other food items online (Hua and Shaw, 2020; Zhang and Ma, 2020). This is mainly a result of the growing deployment of digital technologies across the country in an attempt to limit interactions among people and mitigate the spread of the virus. Secondly, subsequent studies must examine the factors affecting consumer comfort within the online world. It is projected that in the next decade, online platforms will transform people’s purchasing behaviors, especially with regards to acquiring food items. Thus, studies are needed to help businesses identify the existing opportunities and mitigate the main threats likely to undermine growth within the online food ordering business. Last but not least, more detailed academic investigation and practical development of payment mechanisms are needed. By its nature, payment mechanisms deal with technological development of payment methods and techniques that constantly try to improve user convenience and experiences of payments. Hence, existing discussions/examinations relied heavily on technical aspects of payment mechanisms (or schemes). However, technologies in business world can generate implications beyond technical dimension, but also in the social, cultural, psychological, and/or even political dimensions (e.g., Yang et al., 2012; Koenig-Lewis et al., 2015; Nelms et al., 2017; Verhoef et al., 2019). Hence, interdisciplinary works, either conceptual or empirical, can contribute to the literature for analyzing on more complex dynamics of online payment – not just about the technology/system *per se*, but also about the ecosystem composed of human, system, and knowledge in it. In sum, the discussions in this section emphasize the importance of business models with high-quality finance (e.g., payment) systems. This article makes the following proposition.

***Proposition 3:*** *A business model with sound finance systems becomes the core of a healthy online food ecosystem.*

### Online-Offline Interactions and Transformations

Shopping food online is viewed by most researchers as one of the biggest disruptions in the supermarket and grocery business models. From smaller stores to fewer discounts and more service and robots, these are just a few of the changes brought about by online platforms (Kuss and Griffiths, 2011). The problem is that few studies are examining whether new disruptions will continue emerging or whether the online food sector has reached maturity. Such studies are necessary because they will help manufacturers and retailers react accordingly. These studies can focus on trying to understand how consumers can purchase food in the future, which can be online or in physical stores or from larger or smaller stores. Some of the research questions can focus on establishing whether future customers will continue buying to take dine at home or consume right on the spot.

Despite the numerous uncertainties, with regards to brick-and-mortar stores, Burgoine et al. (2017) note that they may survive even with the growth and prevalence of online businesses. As such, future studies must explore how changes in e-commerce will affect shoppers and online businesses. Such studies are essential because the current findings on consumer behavior seem to suggest that customers prefer interacting at a physical store by seeing, smelling, and even touching products they find there. Moreover, there is an immediate satisfaction when a customer picks up something. The insights generated from such studies can help retailers establish the inherent need to focus their attention on emotional elements as well as create unique experiences.

Studies focused on making future forecasting will help in understanding how online food platforms can achieve the social roles enjoyed by supermarkets. Otten et al. (2017) note that supermarkets increasingly place their shopper firsts and tap into their individual needs in an attempt to mitigate the rising competition from online shopping. As such, studies must thoroughly analyze the existing demographic data to make future predictions on whether the online food ordering platforms can ever enjoy the same social roles which are currently the precincts of supermarkets. Finally, a sufficient number of studies have predicted that artificial intelligence and robots are likely to take over the responsibilities of human beings within the online food sector. However, while most of these studies make future predictions, they do not take into account how automation and artificial intelligence will help online supermarkets to become more efficient. Thus, subsequent studies should establish a balance between human interaction and automation. This article makes the following proposition according to the discussions here.

***Proposition 4:*** *The interaction and transformation between online (virtual) and offline (virtual) food businesses determines the dynamic development of future food shopping.*

## Conclusion

The majority of studies examining online food shopping have provided sufficient evidence highlighting both the implications and benefits of e-commerce. However, most of these studies generalize all forms of online shopping and ignore the fact that shopping foods online is inherently different from buying other commodities. As such, the comprehensive academic review conducted helps at explicating the significant themes within the current literature. Hence, the critical propositions that reflected from these studies help in proposing the following four future research directions. They include conducting studies to highlight the customer and business’ perspectives, making future predictions, understanding the implications of ordering via mobile apps, and examining the emerging technologies in online food ordering. The academic review and prepositions made are significant to both researchers and online food stores as people across the world start embracing online shopping more than ever before.

### Theoretical Implications

To generate theoretical implications in a more holistic and comprehensive level, this article focuses on the inter-relationships between the four propositions derived after our conceptual analysis. To recall, the four propositions are inherently about: engaging stakeholders to co-create values, in-depth understanding of technological natures, well-designed business models and finance systems, and online-offline dynamics. One suggestion for future research directions is to develop a holistic-view, often qualitative investigation of a online food shopping ecosystem that composes of interested stakeholders operating with diverse technological sets embedded in well-designed business models that simultaneously incorporate concerns of both online and offline developments of food shopping. Complexity is a point to be explored but is often oversimplified if we could not take a eco-systematic perspective and analyze for both qualitative-quantitative data sources. For a better theoretical development and practical design, the complexity of a food shopping ecosystem can help identify research questions, sketch phenomenon structures and elements, as well as specify heterogeneous interests for policy making. Following this point, another suggestion for future research directions is to address established issues/research questions through cross-disciplinary explorations. As has been discussed, complexity characterizes modern food shopping system, especially the online one. To explore in-depth knowledge of complexity, single disciplinary system of thoughts might limit the imaginations one can create. A cross-discipline approach for studies on online food shopping can both offer fresh explanations for unanswered questions or that in tension, and also help identifying unnoticed phenomenon for further exploration.

### Practical Implications

For online retailers, conceptual analyses and the four resulting propositions can generate practical implications too. First, when designing a online food shopping business/system, practitioners need to adopt an ecosystem viewpoint to prevent incomplete thinking and ignorance of any stakeholder’s opinion. Second, practitioners need to take care of the interfaces between the virtual and physical sub-systems even if it is an online food shopping ecosystem. By considering the interfaces between the sub-systems, not just connection and coordination works would be cared about, but also transformation work should be articulated. For example, the transformation of values in the process flows between material (e.g., food products), informational/technological (safety labels; blockchain applications in supply chain communications; human-machines interface in online purchase procedures, etc.), financial (budgeting; pricing; payment, etc.), human (i.e., stakeholders), and other sub-systems should be implemented with a fully consistent and engaging logic.

### Limitations

In nature, a conceptual analysis is done without empirical and original data collection. However, this article has tried to avoid such inherent limitation by conducting the conceptual analysis with as many practical examples as possible. Additionally, our analysis focuses on the online shopping for foods only. Future studies can also take a similar approach but discuss other characterized industries, such as online shopping for precious metals, intangible services, and so on. Also, our focus on food is limited to foods in general. Future studies can be more detailed, by characterizing more for different food categories (e.g., organic vs. non-organic foods).

## Author Contributions

C-FL was the major author of this article. C-HL reviewed and revised the manuscript. Both authors contributed to the article and approved the submitted version.

## Conflict of Interest

The authors declare that the research was conducted in the absence of any commercial or financial relationships that could be construed as a potential conflict of interest.
